# Temporal trends in cardiovascular burden among patients with prostate cancer receiving androgen deprivation therapy: a population-based cohort study

**DOI:** 10.1038/s41416-023-02271-5

**Published:** 2023-04-19

**Authors:** Jeffrey Shi Kai Chan, Danish Iltaf Satti, Yan Hiu Athena Lee, Jeremy Man Ho Hui, Edward Christopher Dee, Kenrick Ng, Kang Liu, Gary Tse, Chi Fai Ng

**Affiliations:** 1 Cardio-Oncology Research Unit, Cardiovascular Analytics Group, Hong Kong—United Kingdom—China collaboration, Hong Kong, China; 2 Division of Urology, Department of Surgery, Faculty of Medicine, The Chinese University of Hong Kong, Hong Kong, China; 3 Department of Radiation Oncology, Memorial Sloan Kettering Cancer Center, New York, NY USA; 4 Department of Medical Oncology, University College London Hospitals NHS Foundation Trust, London, UK; 5 Tianjin Key Laboratory of Ionic-Molecular Function of Cardiovascular Disease, Department of Cardiology, Tianjin Institute of Cardiology, Second Hospital of Tianjin Medical University, 300211 Tianjin, China; 6 Kent and Medway Medical School, Canterbury, Kent, CT2 7NT UK; 7 School of Nursing and Health Studies, Hong Kong Metropolitan University, Hong Kong, China; 8 SH Ho Urology Centre, The Chinese University of Hong Kong, Hong Kong, China

**Keywords:** Outcomes research, Prostate cancer, Epidemiology

## Abstract

**Background:**

Although androgen deprivation therapy (ADT) is associated with cardiovascular risks, the extent and temporal trends of cardiovascular burden amongst patients with prostate cancer receiving ADT are unclear.

**Methods:**

This retrospective cohort study analyzed adults with PCa receiving ADT between 1993–2021 in Hong Kong, with follow-up until 31/9/2021 for the primary outcome of major adverse cardiovascular events (MACE; composite of cardiovascular mortality, myocardial infarction, stroke, and heart failure), and the secondary outcome of mortality. Patients were stratified into four groups by the year of ADT initiation for comparisons.

**Results:**

Altogether, 13,537 patients were included (mean age 75.5 ± 8.5 years old; mean follow-up 4.7 ± 4.3 years). More recent recipients of ADT had more cardiovascular risk factors and used more cardiovascular or antidiabetic medications. More recent recipients of ADT had higher risk of MACE (most recent (2015–2021) vs least recent (1993–2000) group: hazard ratio 1.33 [1.11, 1.59], *P* = 0.002; *P*_trend_ < 0.001), but lower risk of mortality (hazard ratio 0.76 [0.70, 0.83], *P* < 0.001; *P*_trend_ < 0.001). The 5-year risk of MACE and mortality for the most recent group were 22.5% [20.9%, 24.2%] and 52.9% [51.3%, 54.6%], respectively.

**Conclusions:**

Cardiovascular risk factors were increasingly prevalent amongst patients with prostate cancer receiving ADT, with increasing risk of MACE despite decreasing mortality.

## Introduction

With an estimated 1.4 million new cases and over 375,000 deaths, prostate cancer (PCa) was the third most common cancer globally in 2020 [1]. Androgen deprivation therapy (ADT), which involves the use of pharmacological agents or surgery to suppress the levels of testosterone, is one of the key components of PCa therapy [2, 3]. Though proven efficacious for PCa treatment, research in recent decades, starting with the groundbreaking work by Keating et al. [4], has demonstrated an increasingly established link between ADT and adverse cardiovascular effects, including increased risks of cardiovascular mortality, non-fatal cardiovascular diseases, myocardial infarction, and stroke [5]. Given the rising prevalence of PCa globally and cardiovascular diseases being the leading cause of non-cancer death amongst PCa patients [6–8], ADT-related adverse cardiovascular effects have become increasingly important.

Nonetheless, there is a paucity of studies characterizing the magnitude of cardiovascular burden amongst patients with PCa receiving ADT, despite the large number of studies having compared ADT to non-ADT treatments or between specific types of ADT [5]. Furthermore, it is unclear whether the cardiovascular burden of these patients has evolved over time. It would have been reasonable to hypothesize that the significant progress made in our understanding of ADT-related adverse cardiovascular effects has significantly influenced the cardiovascular outcome of these patients. As such, this study aimed to describe the cardiovascular burden amongst patients with PCa receiving ADT and explore the temporal trends in such burden over the past decades.

## Methods

This retrospective cohort study was performed in accordance with the Declaration of Helsinki and the Strengthening the Reporting of Observational Studies in Epidemiology (STROBE) guideline, and was approved by the Joint Chinese University of Hong Kong—New Territories East Cluster Clinical Research Ethics Committee. The requirement for patient consent has been waived due to the use of deidentified data. All underlying data is available on reasonable request to the corresponding authors.

### Source of data

Data were obtained from the Clinical Data Analysis and Reporting System (CDARS), a population-based, administrative electronic medical records database of all patients attending public healthcare institutions in Hong Kong which serve an estimated 90% of the population [9]. Diagnoses were recorded by *International Classification of Diseases, Ninth revision* (ICD-9) codes regardless of the time of data entry, as CDARS has not implemented ICD-10 codes to date. Mortality data were obtained from the linked Hong Kong Death Registry, a governmental database containing the death record of all Hong Kong citizens, in which the cause of death is recorded using ICD-9 or ICD-10 codes. CDARS and the Hong Kong Death Registry have been used extensively in previous studies and shown to have good coding accuracy [10–15].

### Patient population

Patients aged 18 years old or above with PCa who received any ADT (medical castration or bilateral orchidectomy (BO)) between January 1, 1999 and March 31, 2021 were analysed.

Patients with a prior history of myocardial infarction (MI), stroke, or heart failure (HF) were excluded from all analyses of the primary outcome. These patients were excluded for two reasons. Firstly, patients with prior occurrence of MI, stroke, or HF were likely to have further recorded attendances to follow up on these conditions, and as all diagnoses were extracted using ICD-9 codes, it would have been difficult to reliably distinguish follow-up attendances from true, recurrent occurrences of events. Second, patients with prior occurrence of MI, stroke, or HF likely had varying severity or multiple prior occurrences of these events, both of which would have caused substantial increase in the heterogeneity of their cardiovascular risks that were difficult to account for. Therefore, excluding these patients avoided this heterogeneity and allowed a better reflection of the cardiovascular risks associated with ADT.

### Data collected

The following baseline variables were collected: age, type of ADT (medical castration or bilateral orchiectomy; medical castration included usage of leuprorelin, triptorelin, goserelin, or degarelix, as other gonadotropin-releasing hormone agonists and antagonists were not available in Hong Kong during the study period), duration of castration, comorbidities (hypertension, diabetes mellitus, dyslipidaemia, chronic kidney disease, chronic liver disease, stroke, MI, ischaemic heart disease, HF, anaemia, atrial fibrillation, ventricular tachyarrhythmia, chronic obstructive pulmonary disease, and known malignancy), use of medications or prior procedures (radiotherapy, chemotherapy, radical prostatectomy, angiotensin-converting enzyme inhibitor or angiotensin receptor blocker, beta-blocker, dihydropyridine calcium channel blockers, non-dihydropyridine calcium channel blockers, metformin, sulphonylurea, dipeptidyl peptidase-4 inhibitors, glucagon-like peptide-1 receptor agonist, insulin, antiplatelet, anticoagulant, corticosteroid, percutaneous coronary intervention, and coronary artery bypass graft), the number of cardiovascular medications being used at baseline, and the number of diabetic medications being used at baseline. Patients were also recorded for ever receiving radiotherapy, chemotherapy, radical prostatectomy, and any androgen receptor signalling inhibitor (ARSI; prescriptions of first-generation ARSIs (flutamide and bicalutamide) and second-generation ARSIs (abiraterone and enzalutamide) were recorded separately; other ARSIs were not available in Hong Kong during the study period). Additionally, the number of patients with available baseline records (within 3 years prior to index date which is the date of ADT initiation) of serum total cholesterol level, high-density lipoprotein cholesterol (HDL-C) level, and haemoglobin A1c (HbA1c) level were recorded, and these variables were described for patients with available records. All comorbidities were identified using ICD-9 codes, which were listed in Supplementary Table 1.

### Follow-up and outcome

All patients were followed up from the date of ADT initiation until September 31, 2021. The primary outcome was major adverse cardiovascular events (MACE), which was defined as the first occurrence of cardiovascular mortality, MI, stroke, or HF. The secondary outcome was all-cause mortality. MI, stroke, and HF were identified by ICD-9 codes as listed in Supplementary Table 1. The cause of death was identified by ICD-9 or ICD-10 codes as listed in Supplementary Table 2.

### Statistical analysis

Continuous variables were expressed as mean ± standard deviation. The cohort was subdivided into four groups by the year of ADT initiation, i.e. 1993–2000, 2001–2007, 2008–2014, and 2015–2021. All baseline characteristics were described for patients in each group. Continuous variables were compared between groups using one-way analysis of variance (ANOVA), while categorical variables were compared using Chi-square test. Between-group trends were tested using the Wilcoxon-type test developed by Cuzick [16]. Due to the nature of the database, missing values could only exist for laboratory test results. The number of patients with missing values were described, and only non-missing values were included in between-group comparisons without any imputation.

Kaplan–Meier incidence curves were constructed to visualize the cumulative incidence of the outcomes for each group. As the group of patients with the most recent ADT initiation (i.e. the 2015–2021 group) could be expected to have the shortest follow-up duration, several approaches were deployed to compare the outcomes between groups. First, incidence rates of the outcomes were calculated for each group with the follow-up duration of the groups restricted to the longest follow-up duration of the 2015–2021 group. The incidence rates (IR) were then compared against that of the group with the earliest ADT initiation (i.e. the 1993–2000 group) using the Mantel–Haenszel method with calculation of the corresponding incidence rate ratios (IRR), and the trends in incidence rates between groups were tested using the log-linear trend test. These analyses on IR and IRR were also repeated without any restriction on the follow-up duration. Second, survival analysis was conducted with the aforementioned restriction on follow-up duration. As no important violation of the proportional hazards assumption was found by the Schoenfield’s residuals-based test and visual inspection of the log-log plot and Kaplan–Meier plot, univariable Cox regression analysis was used to compare the cumulative incidence of the outcomes between groups with the 1993–2000 group as reference, and with hazard ratio (HR) and 95% confidence interval (CI) as summary statistics. Third, the 5-year risk of the outcomes were estimated for each group using life tables.

Two sensitivity analyses were performed. First, the restricted mean survival time (RMST) for each group were calculated. Second, as some patients died without having MACE, non-cardiovascular mortality was a competing event for MACE. Hence, univariable competing risk regression was performed for MACE using the Fine and Gray sub-distribution model with non-cardiovascular mortality as the competing event; sub-hazard ratio (SHR) and 95% CI were used as summary statistics.

Lastly, to better understand the risk factors for the outcomes, all baseline variables except laboratory test results were entered for backward stepwise Cox regression with *P* ≥ 0.10 as the threshold for removal and *P* < 0.05 as the threshold for entry. The year of ADT initiation was also entered as a categorical variable to account for any temporal difference in the outcomes. This also accounted for the temporal difference in follow-up duration; thus, no restriction was placed on the follow-up duration in this analysis.

All *P* values were two-sided, with *P* < 0.05 considered statistically significant. All statistical analyses were performed on Stata v16.1 (StataCorp LLC, College Station, Texas, USA).

## Results

In total, 13,537 patients were identified and analysed (mean age 75.5 ± 8.5 years old). Baseline characteristics of included patients were summarized in Table 1. There was a trend favouring medical castration and against BO in more recent years (85.8% with medical castration and 24.4% with BO in 2015–2021 group vs 11.1% with medical castration and 91.2% with BO in 1993–2000 group; Chi-square *P* < 0.001 and *P*_trend_ < 0.001 for both). Fewer of those receiving ADT in more recent years had received radiotherapy (2.5% in 2015–2021 group vs 6.1% in 1993–2000 group, Chi-square *P* < 0.001, *P*_trend_ < 0.001) or radical prostatectomy (19.4% in 2015–2021 group vs 47.3% in 1993–2000 group, Chi-square *P* < 0.001, *P*_trend_ < 0.001). Meanwhile, more of those who received ADT in more recent years have ever received first-generation (44.4% in 2015–2021 group vs 8.4% in 1993–2000 group, Chi-square *P* < 0.001, *P*_trend_ < 0.001) or second-generation (19.8% in 2015–2021 group vs 0.3% in 1993–2000 group, Chi-square *P* < 0.001, *P*_trend_ < 0.001) ARSI, as well as chemotherapy (16.1% in 2015–2021 group vs 0.8% in 1993–2000 group, Chi-square *P* < 0.001, *P*_trend_ < 0.001). Amongst those who received medical castration, the duration of medical castration was shorter in the most recent group (596 ± 564 days in 2015–2021 group vs 974 ± 1409 days in 1993–2000 group, ANOVA *P* < 0.001, *P*_trend_ < 0.001), although this may have been due to the shorter follow-up duration inherent to this group.

**Table 1:** Characteristics for all patients and stratified by the year of androgen deprivation therapy initiation.

		All patients	1993–2000	2001–2007	2008–2014	2015–2021	*P* value between groups	*P* for trend
Number of patients, *N*		13,537	1134	3017	4641	4745	NA	NA
Follow-up duration, years		4.7 ± 4.3	5.7 ± 6.6	6.5 ± 5.6	5.4 ± 3.7	2.6 ± 1.7	<0.001	<0.001
Age, years		75.5 ± 8.5	74.9 ± 7.8	75.2 ± 7.8	75.7 ± 8.5	75.8 ± 9.0	<0.001	<0.001
Medical castration, *N* (%)		8178 (60.4)	126 (11.1)	1213 (40.2)	2768 (59.6)	4071 (85.8)	<0.001	<0.001
Bilateral orchidectomy, *N* (%)		6593 (48.7)	1034 (91.2)	2075 (68.8)	2326 (50.1)	1158 (24.4)	<0.001	<0.001
Duration of ADT, days^a^		891 ± 905	842 ± 1160	1066 ± 1287	1116 ± 1026	687 ± 558	<0.001	<0.001
Hypertension, *N* (%)		3624 (26.8)	123 (10.9)	549 (18.2)	1293 (27.9)	1659 (35.0)	<0.001	<0.001
Diabetes mellitus, *N* (%)		2886 (21.3)	87 (7.7)	484 (16.0)	1014 (21.9)	1301 (27.4)	<0.001	<0.001
Dyslipidaemia, *N* (%)		1270 (9.4)	9 (0.8)	97 (3.2)	364 (7.8)	800 (16.9)	<0.001	<0.001
Chronic kidney disease, *N* (%)		452 (3.3)	18 (1.6)	70 (2.3)	177 (3.8)	187 (3.9)	<0.001	<0.001
Chronic liver disease, *N* (%)		146 (1.1)	5 (0.4)	14 (0.5)	56 (1.2)	71 (1.5)	<0.001	<0.001
Stroke, *N* (%)		1216 (9.0)	42 (3.7)	221 (7.3)	424 (9.1)	529 (11.2)	<0.001	<0.001
Myocardial infarction, *N* (%)		427 (3.2)	9 (0.8)	73 (2.4)	156 (3.4)	189 (4.0)	<0.001	<0.001
Ischaemic heart disease, *N* (%)		1407 (10.4)	76 (6.7)	260 (8.6)	507 (10.9)	564 (11.9)	<0.001	<0.001
Heart failure, *N* (%)		695 (5.1)	35 (3.1)	116 (3.8)	278 (6.0)	266 (5.6)	<0.001	<0.001
Anaemia, *N* (%)		966 (7.1)	34 (3.0)	112 (3.7)	418 (9.0)	402 (8.5)	<0.001	<0.001
Atrial fibrillation, *N* (%)		610 (4.5)	24 (2.1)	90 (3.0)	212 (4.6)	284 (6.0)	<0.001	<0.001
COPD, *N* (%)		804 (5.9)	53 (4.7)	193 (6.4)	316 (6.8)	242 (5.1)	0.001	0.341
Known malignancy, *N* (%)		1003 (13.3)	130 (11.5)	403 (13.4)	701 (15.1)	569 (12.0)	<0.001	0.558
Prior PCI, *N* (%)		432 (3.2)	6 (0.5)	47 (1.6)	157 (3.4)	222 (4.7)	<0.001	<0.001
Prior CABG, *N* (%)		55 (0.4)	1 (0.1)	10 (0.3)	17 (0.4)	27 (0.6)	0.088	0.015
Prior radiotherapy, *N* (%)		493 (3.6)	69 (6.1)	105 (3.5)	199 (4.3)	120 (2.5)	<0.001	<0.001
Prior RP, *N* (%)		3735 (27.6)	536 (47.3)	1071 (35.5)	1206 (26.0)	922 (19.4)	<0.001	<0.001
Prior chemotherapy, *N* (%)		61 (0.5)	0 (0)	3 (0.1)	11 (0.2)	47 (1.0)	<0.001	<0.001
Ever received radiotherapy, *N* (%)		3114 (23.0)	328 (28.9)	799 (26.5)	1294 (27.9)	693 (14.6)	<0.001	<0.001
Ever received RP, *N* (%)		4601 (34.0)	603 (53.2)	1286 (42.6)	1456 (31.4)	1256 (26.5)	<0.001	<0.001
Ever received chemotherapy, *N* (%)		1311 (9.7)	9 (0.8)	121 (4.0)	416 (9.0)	765 (16.1)	<0.001	<0.001
Ever received first-generation ARSI, *N* (%)		4239 (31.5)	92 (8.4)	663 (22.1)	1377 (29.7)	2107 (44.4)	<0.001	<0.001
Ever received second-generation ARSI, *N* (%)		1582 (11.7)	3 (0.3)	77 (2.6)	563 (12.1)	939 (19.8)	<0.001	<0.001
Ever received chemotherapy or ARSI, *N* (%)		5116 (37.8)	101 (8.9)	724 (24.0)	1638 (35.3)	2653 (55.9)	<0.001	<0.001
Number of cardiovascular medications		1.5 ± 1.7	0.2 ± 0.6	1.0 ± 1.3	1.7 ± 1.6	2.0 ± 1.8	<0.001	<0.001
Number of antidiabetic medications		0.30 ± 0.73	0.05 ± 0.28	0.21 ± 0.57	0.32 ± 0.74	0.40 ± 0.85	<0.001	<0.001
ACEI/ARB users, *N* (%)		3383 (25.0)	23 (2.0)	481 (15.9)	1303 (28.1)	1576 (33.2)	<0.001	<0.001
Beta-blocker users, *N* (%)		4130 (30.5)	42 (3.7)	696 (23.1)	1638 (35.3)	1754 (37.0)	<0.001	<0.001
Dihydropyridine CCB users, *N* (%)		5396 (39.9)	58 (5.1)	886 (29.4)	2058 (44.3)	2394 (50.5)	<0.001	<0.001
Non-dihydropyridine CCB users, *N* (%)		575 (4.3)	11 (1.0)	134 (4.4)	226 (4.9)	201 (4.3)	<0.001	0.002
Metformin users, *N* (%)		1480 (10.9)	14 (1.2)	194 (6.4)	584 (12.6)	688 (14.5)	<0.001	<0.001
Sulphonylurea users, *N* (%)		1744 (12.9)	29 (2.6)	347 (11.5)	661 (14.2)	707 (14.9)	<0.001	<0.001
DPP4 inhibitor users, *N* (%)		150 (1.1)	0 (0)	0 (0)	29 (0.6)	121 (2.6)	<0.001	<0.001
GLP1 receptor agonist users, *N* (%)		2 (0.0)	0 (0)	0 (0)	1 (0.0)	1 (0.0)	0.829	0.423
Insulin users, *N* (%)		722 (5.3)	10 (0.9)	90 (3.0)	222 (4.8)	400 (8.4)	<0.001	<0.001
Antiplatelet users, *N* (%)		2962 (21.9)	43 (3.8)	531 (17.6)	1112 (24.0)	1276 (27.9)	<0.001	<0.001
Anticoagulant users, *N* (%)		458 (3.4)	3 (0.3)	58 (1.9)	148 (3.2)	249 (5.3)	<0.001	<0.001
Corticosteroid users, *N* (%)		2342 (17.3)	29 (2.6)	510 (16.9)	933 (20.1)	870 (18.3)	<0.001	<0.001
With available total cholesterol, HDL-C, and HbA1c levels, *N* (%)		3940 (29.1)	5 (0.4)	265 (8.8)	1220 (26.3)	2450 (51.6)	<0.001	<0.001
Total cholesterol	Available, *N* (%)	6727 (49.7)	28 (2.5)	806 (26.7)	2458 (53.0)	3435 (72.4)	<0.001	<0.001
	Level, mmol/L	4.41 ± 1.01	5.25 ± 2.20	4.79 ± 0.95	4.56 ± 1.01	4.21 ± 0.96	<0.001	<0.001
HDL-C	Available, *N* (%)	6478 (47.9)	15 (1.3)	626 (20.8)	2415 (52.0)	3422 (72.1)	<0.001	<0.001
	Level, mmol/L	1.27 ± 0.37	1.17 ± 0.31	1.28 ± 0.37	1.27 ± 0.37	1.28 ± 0.37	0.335	0.299
HbA1c	Available, *N* (%)	4295 (31.7)	22 (1.9)	403 (13.4)	1320 (28.4)	2550 (53.7)	<0.001	<0.001
	Level, %	6.49 ± 1.16	7.85 ± 1.76	6.94 ± 1.46	6.64 ± 1.18	6.33 ± 1.06	<0.001	<0.001

*ACEI* angiotensin-converting enzyme inhibitor, *ARB* angiotensin receptor blocker, *ARSI* androgen receptor signaling inhibitor, *CABG* coronary artery bypass graft, *CCB* calcium channel blocker, *COPD* chronic obstructive pulmonary disease, *DPP4* dipeptidyl peptidase-4, *GLP1* glucagon-like peptide-1, *HbA1c* haemoglobin A1c, *HDL-C* high-density lipoprotein cholesterol, *NA* not applicable, *RP* radical prostatectomy.

^a^Only including patients who received medical castration

A total of 2059 (15.2%) had a prior diagnosis of MI, stroke, or HF, and were thus excluded from all analyses of MACE, resulting in a sample size of 11,478 patients for the MACE analyses. Over a mean follow-up duration of 4.7 ± 4.3 years, 2727 patients (23.8%) had MACE, and 9124 (67.4) died. Kaplan–Meier curves showing the cumulative incidence of MACE and all-cause mortality without any restriction on follow-up durations were shown in Supplementary Figs. 1 and 2, respectively. As expected, patients with the most recent ADT initiation had the shortest follow-up duration, with the longest observed follow-up duration being 6.7 years.

### Cardiovascular risk factors and medications

Overall, patients who were initiated on ADT more recently had more cardiovascular risk factors (Table 1), with higher rates of prior hypertension, diabetes mellitus, stroke, myocardial infarction, ischaemic heart, heart failure, chronic kidney disease, atrial fibrillation, and dyslipidaemia. These patients were also slightly older. Correspondingly, more of those who had ADT initiated more recently were using cardiovascular or antidiabetic medications at baseline. Also, more of those who had ADT initiated more recently had received percutaneous coronary intervention.

Additionally, more of those who had ADT initiated recently had been checked for levels of total cholesterol, HDL-C, and HbA1c prior to initiating ADT. This was accompanied by lower total cholesterol and HbA1c amongst those with available test results whose ADT was initiated recently, but without any significant differences in HDL-C levels. Overall, despite an increasing proportion of patients having all three laboratory markers profiled prior to ADT initiation, only 51.6% of the 2015–2021 group had all three markers profiled (vs 0.4% in 1993–2000 group, Chi-square *P* < 0.001, *P*_trend_ < 0.001).

### Major adverse cardiovascular event

With follow-up durations restricted to the longest observed follow-up duration of the 2015–2021 group (6.7 years), patients initiated on ADT more recently had higher IR of MACE (IR 5.7 [5.3, 6.2] events per 100 person-year in the 2015–2021 group vs 4.4 [95% CI 3.7, 5.1] events per 100 person-year in the 1993–2000 group; IRR 1.32 [1.10, 1.58], *P* = 0.003 for the 2015–2021 group compared to the 1993–2000 group; log-linear trend test *P* < 0.001; Table 2). Similar observations were made with IR and IRR calculated without any restriction on follow-up durations (Supplementary Table 3).

**Table 2:** Incidence rates of both outcomes with the follow-up duration restricted to the longest observed follow-up duration of the 2015–2021 group (6.7 years), stratified by the year of androgen deprivation therapy initiation.

Year of androgen deprivation therapy initiation	Major adverse cardiovascular events		All-cause mortality	
	Incidence rate^a^	Incidence rate ratio	Incidence rate^a^	Incidence rate ratio
1993–2000	4.4 [3.7, 5.1]	1 (reference)	20.3 [19.0, 21.8]	1 (reference)
2001–2007	4.7 [4.3, 5.1]	1.07 [0.89, 1.28], *P* = 0.479	14.5 [13.9, 15.2]	0.71 [0.66, 0.78], *P* < 0.001
2008–2014	5.4 [5.0, 5.7]	1.23 [1.04, 1.47], *P* = 0.019	13.9 [13.4, 14.5]	0.69 [0.63, 0.74], *P* < 0.001
2015–2021	5.7 [5.3, 6.2]	1.32 [1.10, 1.58], *P* = 0.003	15.9 [15.2, 16.6]	0.78 [0.72, 0.85], *P* < 0.001

^a^Per 100 person-year.

Incidence rate ratios displayed were referenced against the 1993–2000 group.

**Fig. 1:**
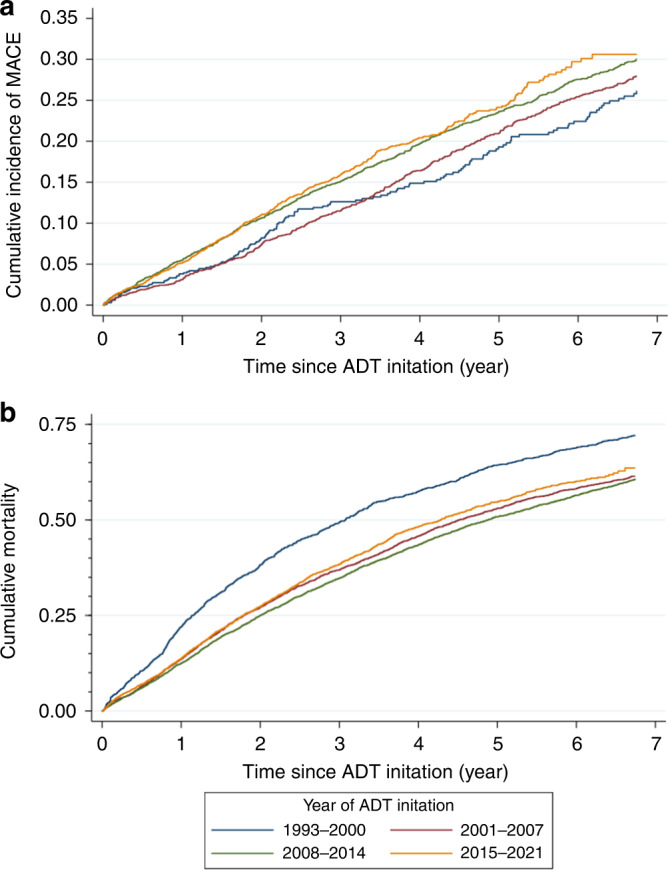
Kaplan–Meier incidence curves. **a** shows the cumulative incidence of major adverse cardiovascular event (MACE), while **b** shows that of all-cause mortality. The follow-up durations in both curves were restircted to the longest observed follow-up duration of the 2015–2021 group (6.7 years). ADT androgen deprivation therapy.

A similar trend was observed in the Cox regression analysis (*P*_trend_ < 0.001), where the patients with ADT initiated in years 2015–2021 had an estimated 33% higher risk of MACE compared to those with ADT initiation in years 1993–2000 (HR 1.33 [1.11, 1.59], *P* = 0.002; Fig. 1a and Table 3). Correspondingly, the estimated 5-year risk of MACE was the highest in the 2015–2021 group (22.5% [20.9%, 24.2%]) and the 2008–2014 group (23.0% [21.6%, 24.5%]), followed by the 2001–2007 group (19.4% [17.7%, 21.1%], and lastly, with the lowest 5-year risk, the 1993–2000 group (17.0% [14.4%, 19.9%]).

**Table 3:** Hazard ratios and accompanying 95% confidence intervals from Cox regression analysis with the follow-up duration restricted to the longest observed follow-up duration of the 2015–2021 group (6.7 years).

Year of androgen deprivation therapy initiation	Major adverse cardiovascular event	All-cause mortality
1993–2000	1 (reference)	1 (reference)
2001–2007	1.07 [0.89, 1.28], *P* = 0.493	0.72 [0.66, 0.78], *P* < 0.001
2008–2014	1.23 [1.03, 1.46], *P* = 0.021	0.69 [0.64, 0.74], *P* < 0.001
2015–2021	1.33 [1.11, 1.59], *P* = 0.002	0.76 [0.70, 0.83], *P* < 0.001

*ADT* androgen deprivation therapy.

Hazard ratios displayed were referenced against the 1993–2000 group.

Sensitivity analyses with RMST demonstrated similar results (RMST 5.59 [5.51, 5.68] years for the 2015–2021 group vs 5.86 [5.73, 6.00] years for the 1993–2000 group; Supplementary Table 4). Competing risk regression also found similar results, with the 2015–2021 group having an estimated 43% higher cumulative incidence of MACE than the 1993–2000 group (SHR 1.43 [1.19, 1.71], *P* < 0.001; Supplementary Table 5).

Backward stepwise Cox regression showed that increased age, diabetes mellitus, hypertension, anaemia, known malignancy, anticoagulant use, and using more cardiovascular medications were independently associated with higher risk of MACE, while statin use, and metformin use were independently associated with lower risk of MACE (Supplementary Table 6).

### All-cause mortality

With follow-up durations restricted to the longest observed follow-up duration of the 2015–2021 group (6.7 years), patients initiated on ADT more recently had lower IR of all-cause mortality (IR 15.9 [15.2, 16.6] deaths per 100 person-year in the 2015–2021 group vs 20.3 [19.0, 21.8] deaths per 100 person-year in the 1993–2000 group; IRR 0.78 [0.72, 0.85], *P* < 0.001; for the 2015–2021 group compared to the 1993–2000 group; log-linear trend test *P* = 0.003; Table 2). However, analysis without any restriction on follow-up duration showed no significant difference between the 2015–2021 group and 1993–2000 group in the IR of all-cause mortality (IRR 0.98 [0.91, 1.06], *P* = 0.660; Supplementary Table 3), which was driven by a markedly lower IR in the 1993–2000 group (IR 16.2 [15.2, 17.2] deaths per 100 person-year when analyzed without any restriction on follow-up duration vs 20.3 [19.0, 21.8] deaths per 100 person-year when analyzed with restricted follow-up duration). Such difference likely resulted from the mortality rate being higher in the initial years after ADT initiation before levelling off in later years, as apparent from the Kaplan–Meier curve without any restriction on follow-up duration (Supplementary Fig. 2).

Cox regression with restricted follow-up duration showed that patients with ADT initiated more recently had lower risk of mortality (*P*_trend_ < 0.001), with the 2015–2021 group having an estimated 24% lower risk of all-cause mortality than those in the 1993–2000 group (HR 0.76 [0.70, 0.83], *P* < 0.001; Fig. 1b and Table 3). The estimated 5-year risk of all-cause mortality was 64.5% [61.7%, 67.4%] for the 1993–2000 group, 53.0% [51.2%, 54.8%] for the 2001–2007 group, 50.8% [49.4%, 52.3%] for the 2008–2014 group, and 52.9% [51.3%, 54.6%] for the 2015–2021 group.

Sensitivity analysis with RMST showed similar results as Cox regression (RMST 3.55 [3.40, 3.70] years for the 1993–2000 group vs 4.14 [4.06, 4.22] years for the 2015–2021 group; Supplementary Table 4).

Backward stepwise Cox regression showed that increased age, diabetes mellitus, anaemia, atrial fibrillation, chronic liver disease, chronic obstructive pulmonary disease, known malignancy, prior chemotherapy, prior radiotherapy, insulin use, sulphonylurea use, and using more cardiovascular medications were independently associated with higher risk of all-cause mortality, while medical castration, statin use, prior radical prostatectomy, angiotensin-converting enzyme inhibitor/angiotensin receptor blocker use, and metformin use were independently associated with lower risk of all-cause mortality (Supplementary Table 7). ADT initiation during or after 2001 was identified to be independently associated with lower risk of all-cause mortality as well. There was a trend of chronic kidney disease being associated with numerically higher risk of all-cause mortality which approached statistical significance (*P* = 0.056).

## Discussion

This population-based study demonstrated that cardiovascular risk factors were increasingly prevalent amongst Asian patients with PCa receiving ADT. This was accompanied by an increasing risk of MACE, despite reducing risk of mortality and an increasing proportion of patients with laboratory profiling relevant to cardiovascular risks.

To the best of our knowledge, this was one of the first studies that systematically quantified the temporal trends of cardiovascular burden in patients with PCa receiving ADT. We observed an increase in the risk of MACE but a decrease in the risk of mortality over time. The latter has been observed in other studies and have been postulated to be due to better treatment of PCa [7]; other factors possibly at play may include changes in lifestyle and access to healthcare over the years. Meanwhile, the former occurred despite better contemporary understanding and recognition of the adverse cardiovascular effects of ADT [5], hence more patients being tested or monitored for cardiovascular risks. While it is immediately apparent that the increasing prevalence of cardiovascular risk factors over time may have contributed significantly to this observation, the factors driving the increase in MACE incidence were likely multifactorial and intertwined. For instance, the increasing use of ARSI and chemotherapy may have contributed to such increase in the risk of MACE, as both classes of agents have been shown to carry significant cardiovascular risks [17], with the newer generation of ARSI having been shown specifically to carry significantly higher cardiovascular risks than conventional ADT [18, 19]. Nonetheless, disentangling these factors, which may further include but are not limited to the patterns of cardiometabolic screening, usage and duration of specific types of ADT, and the stage of disease when PCa was detected or when ADT was initiated, was outside the scope of the current study.

The increasing prevalence of cardiovascular risk may have been a direct result of the well-characterized, general increase in the prevalence of cardiovascular diseases [6]. Systemic factors, such as patient selection for ADT, and patients’ knowledge and perception of ADT, may have played a role also. Indeed, we showed that the preference for specific modalities of ADT changed over time. This change maybe partly related to the change in local medical reimbursement system, as well as an overwhelming preference for medical castration over BO in more recent years which was consistent with survey studies showing an estimated two-third of clinicians not considering or offering BO to eligible patients with PCa [20, 21]. It was possible that similar changes in the selection of patients for ADT have influenced the cardiovascular outcomes. This was especially relevant since the use of ADT is heavily dependent on patient preferences even within guidelines [3, 22], with studies having shown significant variations in practice [23, 24]. It may thus be reasonable to speculate that changes in patient preferences outside the scope of this study may have contributed to the differences in cardiovascular burden and outcomes, either directly or by influencing patient selection for ADT.

Previous studies have shown that cardiovascular risk factors are prevalent amongst patients with PCa. In a large, prospective Canadian cohort of 2492 patients with PCa, 22% had known cardiovascular diseases [25]. Similarly, in another smaller cohort of patients with PCa undergoing ADT, a quarter had established cardiovascular diseases [26]. We observed similar results particularly in the most contemporary subgroup of patients, with 35.0% having hypertension and 27.4% having diabetes mellitus at baseline, and over 11% having had stroke or MI. In addition, we observed that only half of the patients in the most contemporary group had had total cholesterol, HDL-C, and HbA1c profiled prior to ADT initiation, which was despite substantial improvement in such proportions over time. This was echoed by a recent study by Sun and colleagues, who found that only 68.1% of an American cohort of veterans with PCa received comprehensive cardiovascular risk factor assessment [27].

### Direct clinical relevance and further directions

Given the increasing risk of MACE observed in the current study, this study serves as a timely reminder for clinicians to be vigilant in screening and managing cardiovascular burden amongst patients with PCa undergoing ADT. The increasing risk of MACE in spite of increasing metabolic screening prior to initiating ADT showed that it is insufficient to only screen these patients, and that more efforts are required to adequately manage and reduce their cardiometabolic risk, including referral of patients with multiple cardiovascular risk factors to cardiology or cardio-oncology services before initiating ADT. Such multidisciplinary approach has been recommended by the 2022 European Society of Cardiology cardio-oncology guideline [28]. This was not only relevant to urologists and oncologists, but also primary care physicians and cardiologists who may take care of patients with PCa receiving ADT. Furthermore, the observed 5-year risks of MACE and mortality reported in this study should allow clinicians to better inform patients of the risks associated with ADT, thereby facilitating shared decision-making regarding therapeutic options. It is important for clinicians to comprehensively inform patients and involve them actively in such decision-making, as greater involvements have been shown to be associated with lower risks of decision regret and higher health-related quality of life [29]. Additionally, the independent risk factors for MACE and all-cause mortality hereby identified may improve identification of patients who are possibly at higher risk of these events. Given the relatively old mean age and long duration of ADT in this study, and as the duration of ADT may be proportionally associated with the risk of adverse cardiovascular outcomes [30], the current results are the most relevant to elderly patients with PCa who will be receiving long-term ADT, especially medical castration which was the predominant modality of ADT in the most contemporary group of patients in the current study. Although the results may not be as relevant to young, otherwise healthy patients who are to be initiated on short-term ADT, the findings remain a valid reminder for clinicians to be vigilant of cardiovascular risks in patients with PCa undergoing ADT in general.

With this study demonstrating the significant and increasing cardiovascular burden amongst patients with PCa undergoing ADT, there are several gaps in evidence that are more relevant than ever. First, evidence pertinent to the optimal screening strategy for cardiovascular diseases in these patients is scarce. While previous studies have used the Framingham risk score as a surrogate of cardiovascular risk [25–27], its accuracy and validity for this specific patient group has not been adequately explored. This was echoed by the 2022 European Society of Cardiology cardio-oncology guideline [28] which pointed out the lack of cardiovascular risk scores for patients receiving ADT. More macroscopically, it is unclear, and therefore remains at clinicians’ discretion, which patients are in particular need for cardiovascular screening and monitoring. To this end, prognostic studies, possibly with exploration of novel risk scores, are sorely needed to allow better stratification of high-risk patients; our reported independent risk factors for MACE and all-cause mortality may be a starting point which such studies may reference.

Second, to the best of our knowledge, there has not been any investigation of strategies to mitigate the adverse cardiovascular effects of ADT. Whilst Bhatia and colleagues have proposed a management algorithm for ADT-related adverse cardiovascular effects, the algorithm was based on previous paradigm established for patients with breast cancer, and direct evidence supporting the algorithm or any specific management approach remains lacking [31, 32]. This was in stark contrast to many other classes of antineoplastic medications that causes adverse cardiovascular effects, for which evidence-based management algorithms have been established [33, 34]. Future studies should therefore investigate approaches and therapeutics to mitigate the adverse cardiovascular effects brought by ADT. Screening, risk stratification, and management of cardiovascular risk factors have all been specified as key areas of focus for future research by an American Heart Association scientific statement as well [35].

Last but not least, further studies are required to elucidate the factors driving the observed worsening of cardiovascular outcome in patients with PCa undergoing ADT, which are likely complex, intertwined, and multifactorial in nature. A better understanding of these factors is necessary for stifling further worsening of cardiovascular outcomes in these patients.

### Generalizability, strengths, and limitations

This study included a large cohort of patients from a population-based database, meaning that the findings are representative of real-life practice locally, and likely generalizable to other developed Asian cities. The consistent findings from multiple, different statistical approaches also reinforced the validity of our findings. However, Hong Kong operates a heavily subsidized public healthcare system, from which an estimated 90% of all Hong Kong citizens receive healthcare. Given that healthcare financing structures have significant impact on access to care and the choice of therapeutics [36, 37], the findings of this study may not be directly generalizable to countries with different healthcare financing structures, such as those that are predominantly privatized. Sociodemographic and cultural differences in the population may also affect generalizability of our findings to other countries and regions.

This study had a few limitations. First, the retrospective, observational nature predisposed to residual and unmeasured confounders which may influence findings. Whilst we acknowledge that there were likely unmeasured factors that may have driven our findings, it was not within the scope of this study to disentangle these underlying drivers – we invite the readers and colleagues to further explore this topic indeed. Second, owing to the nature of the database used, cancer staging and some cardiovascular risk factors that are prognostic in cardiovascular diseases, such as blood pressure and smoking status, were not available, which may have been important confounders, and which limited the interpretation of our findings. Specifically, there have been reports showing earlier diagnosis of PCa in recent years [38], which may have contributed to differences in the incidence and risk of both all-cause mortality and MACE – this remains to be investigated in the future. Third, as the data was retrieved from a deidentified, administrative database, it could not be individually adjudicated. Nonetheless, data entry was performed by treating clinicians without the involvement of any of the authors, and none of the authors had the right or authority to alter the data. Furthermore, previous studies have demonstrated that data recorded in the database (CDARS) had good accuracy, particularly for cardiovascular outcomes [39, 40].

## Conclusions

Over the past 29 years, cardiovascular risk factors have become increasingly prevalent amongst patients with PCa receiving ADT in Hong Kong. This was accompanied by an increasing incidence of MACE but a decreasing incidence of all-cause mortality. Factors underlying such observations remain to be elucidated.

## Supplementary information

Supplementary Material

STROBE checklist

## Data Availability

All underlying data is available on reasonable request to the corresponding authors.
