# Supplementary material for: Temporal trends in cardiovascular burden among patients with prostate cancer receiving androgen deprivation therapy: a population-based cohort study
Source: Br J Cancer. 2023 Apr 19;128(12):2253–60. doi: 10.1038/s41416-023-02271-5 (PMC10241887; doi:10.1038/s41416-023-02271-5)

# SUPPLEMENTARY MATERIAL

**Supplementary Table 1.** International Classification of Diseases, Ninth Revision (ICD-9) codes used to identify outcomes and co-morbidities. All hereby listed codes include the corresponding sub-codes.

| | | | | | | | | | | | | | | |
|---|---|---|---|---|---|---|---|---|---|---|---|---|---|---|
| Prostate cancer | 185 | | | | | | | | | | | | | |
| Heart failure | 428 | | | | | | | | | | | | | |
| Myocardial infarction | 410 | | | | | | | | | | | | | |
| Diabetes mellitus | 250 | | | | | | | | | | | | | |
| Hypertension | 401 | 402 | 403 | 404 | 405 | 437.2 | | | | | | | | |
| Atrial fibrillation | 427.31 | | | | | | | | | | | | | |
| Stroke | 430 | 431 | 432 | 433 | 434 | 435 | | | | | | | | |
| Chronic obstructive pulmonary disease | 490 | 491 | 492 | 496.0 | | | | | | | | | | |
| Ischaemic heart disease | 410 | 411 | 412 | 413 | 414 | | | | | | | | | |
| Chronic kidney disease | 582 | 585 | 586 | | | | | | | | | | | |
| Chronic liver disease | 456.0 | 456.1 | 456.20 | 456.21 | 571 | 572.2 | 572.3 | 572.4 | 572.5 | 572.6 | 572.7 | 572.8 | | |
| Anaemia | 280 | 281 | 282 | 283 | 284.0 | 284.1 | 284.8 | 284.9 | 285 | | | | | |
| Dyslipidaemia | 272.0 | 272.1 | 272.2 | 272.3 | 272.4 | | | | | | | | | |
| Malignancy | 140 | 141 | 142 | 143 | 144 | 145 | 146 | 147 | 148 | 149 | 150 | 151 | 152 | 153 |
| | | 154 | 155 | 156 | 157 | 158 | 159 | 160 | 161 | 162 | 163 | 164 | 165 | 170 |
| | | 171 | 172 | 173 | 174 | 175 | 179 | 179 | 180 | 181 | 182 | 183 | 184 | 185 |
| | | 186 | 187 | 188 | 189 | 190 | 191 | 192 | 193 | 194 | 195 | 196 | 197 | 198 |
| | | 199 | 200 | 201 | 202 | 203 | 204 | 205 | 206 | 207 | 208 | 209.0 | 209.1 | 209.2 |
| | | 209.3 | | | | | | | | | | | | |

**Supplementary Table 2.** International Classification of Diseases, Ninth Revision (ICD-9) and Tenth Revision (ICD-10) codes used to identify the cause of death. All hereby listed codes include the corresponding sub-codes.

| Type of mortality | ICD codes |
|---|---|
| Cardiovascular mortality | ICD-9: 390-438<br>ICD-10: I00-I79 |

**Supplementary Table 3.** Incidence rates of both outcomes without any restriction on the follow-up duration, stratified by the year of androgen deprivation therapy initiation. Incidence rate ratios displayed were referenced against the 1993-2000 group.

| Year of androgen deprivation therapy initiation | Major adverse cardiovascular events [1] | | All-cause mortality [2] | |
|---|---|---|---|---|
| | Incidence rate [3] | Incidence rate ratio | Incidence rate [3] | Incidence rate ratio |
| 1993-2000 | 4.1 [3.6, 4.6] | 1 (reference) | 16.2 [15.2, 17.2] | 1 (reference) |
| 2001-2007 | 5.0 [4.7, 5.4] | 1.23 [1.06, 1.43], p=0.006 | 13.6 [13.1, 14.1] | 0.84 [0.78, 0.91], p<0.001 |
| 2008-2014 | 5.5 [5.2, 5.9] | 1.36 [1.18, 1.57], p<0.001 | 13.8 [13.3, 14.3] | 0.85 [0.80, 0.92], p<0.001 |
| 2015-2021 | 5.7 [5.3, 6.2] | 1.41 [1.21, 1.65], p<0.001 | 15.9 [15.2, 16.6] | 0.98 [0.91, 1.06], p=0.660 |

[1] Log-linear test for trend p<0.001

[2] Log-linear test for trend p=0.168

[3] Per 100 person-year

**Supplementary Table 4.** Restricted mean survival time (in years) for both outcomes, stratified by the year of androgen deprivation therapy initiation. Follow-up duration was restricted to the longest follow-up duration observed in the 2015-2021 group.

| Year of androgen deprivation therapy initiation | Major adverse cardiovascular event | All-cause mortality |
|---|---|---|
| 1993-2000 | 5.86 [5.73, 6.00] | 3.55 [3.40, 3.70] |
| 2001-2007 | 5.82 [5.74, 5.89] | 4.23 [4.14, 4.32] |
| 2008-2014 | 5.64 [5.58, 5.71] | 4.36 [4.29, 4.43] |
| 2015-2021 | 5.59 [5.51, 5.68] | 4.14 [4.06, 4.22] |

**Supplementary Table 5.** Competing risk regression for major adverse cardiovascular event using the Fine and Gray sub-distribution model, with non-cardiovascular mortality as the competing event.

| Year of androgen deprivation therapy initiation | Sub-hazard ratio [95% confidence interval] | P value |
|---|---|---|
| 1993-2000 | 1 (reference) | Not applicable |
| 2001-2007 | 1.30 [1.09, 1.56] | 0.004 |
| 2008-2014 | 1.58 [1.33, 1.88] | <0.001 |
| 2015-2021 | 1.43 [1.19, 1.71] | <0.001 |

**Supplementary Table 6.** Results of backward stepwise Cox regression identifying the independent risk factors of major adverse cardiovascular event.

| | Hazard ratio [95% confidence interval] | P value |
|---|---|---|
| Age (per year) | 1.05 [1.04, 1.05] | <0.001 |
| Diabetes mellitus | 1.41 [1.24, 1.61] | <0.001 |
| Hypertension | 1.18 [1.07, 1.30] | 0.001 |
| Anaemia | 1.36 [1.16, 1.60] | <0.001 |
| Known malignancy | 1.46 [1.29, 1.64] | <0.001 |
| Statin use | 0.82 [0.72, 0.94] | 0.003 |
| Anticoagulant use | 1.55 [1.24, 1.93] | <0.001 |
| Metformin use | 0.74 [0.63, 0.87] | <0.001 |
| Number of cardiovascular medication (per drug item) | 1.17 [1.12, 1.21] | <0.001 |

**Supplementary Table 7.** Results of backward stepwise Cox regression identifying the independent risk factors of all-cause mortality.

| | Hazard ratio [95% confidence interval] | P value |
|---|---|---|
| Age (per year) | 1.04 [1.03, 1.04] | <0.001 |
| Medical castration | 0.83 [0.79, 0.87] | <0.001 |
| Prior chemotherapy | 1.73 [1.20, 2.50] | 0.003 |
| Year of androgen deprivation therapy initiation | | |
| 2001-2007 | 0.81 [0.75, 0.88] | <0.001 |
| 2008-2014 | 0.75 [0.70, 0.81] | <0.001 |
| 2015-2021 | 0.86 [0.78, 0.93] | 0.001 |
| Diabetes mellitus | 1.24 [1.14, 1.36] | <0.001 |
| Insulin use | 1.17 [1.06, 1.30] | 0.003 |
| Chronic kidney disease | 1.12 [1.00, 1.25] | 0.056 |
| Anaemia | 1.63 [1.51, 1.76] | <0.001 |
| Atrial fibrillation | 1.17 [1.06, 1.29] | 0.003 |
| Chronic liver disease | 1.49 [1.22, 1.82] | <0.001 |
| Chronic obstructive pulmonary disease | 1.35 [1.24, 1.45] | <0.001 |
| Prior radiotherapy | 1.17 [1.06, 1.31] | 0.003 |
| Statin use | 0.91 [0.85, 0.98] | 0.015 |
| Known malignancy | 2.04 [1.93, 2.17] | <0.001 |
| Prior radical prostatectomy | 0.92 [0.88, 0.97] | 0.001 |
| ACEI/ARB use | 0.93 [0.87, 0.99] | 0.023 |
| Metformin use | 0.60 [0.54, 0.66] | <0.001 |
| Sulphonylurea use | 1.13 [1.03, 1.24] | 0.013 |
| Number of cardiovascular medication (per drug item) | 1.06 [1.03, 1.08] | <0.001 |

ACEI, angiotensinogen-converting enzyme inhibitor. ARB, angiotensin receptor blocker.

**Supplementary Figure 1.** Kaplan-Meier curve showing the cumulative incidence of major adverse cardiovascular event (MACE) without any restriction on the follow-up duration. ADT, androgen deprivation therapy.

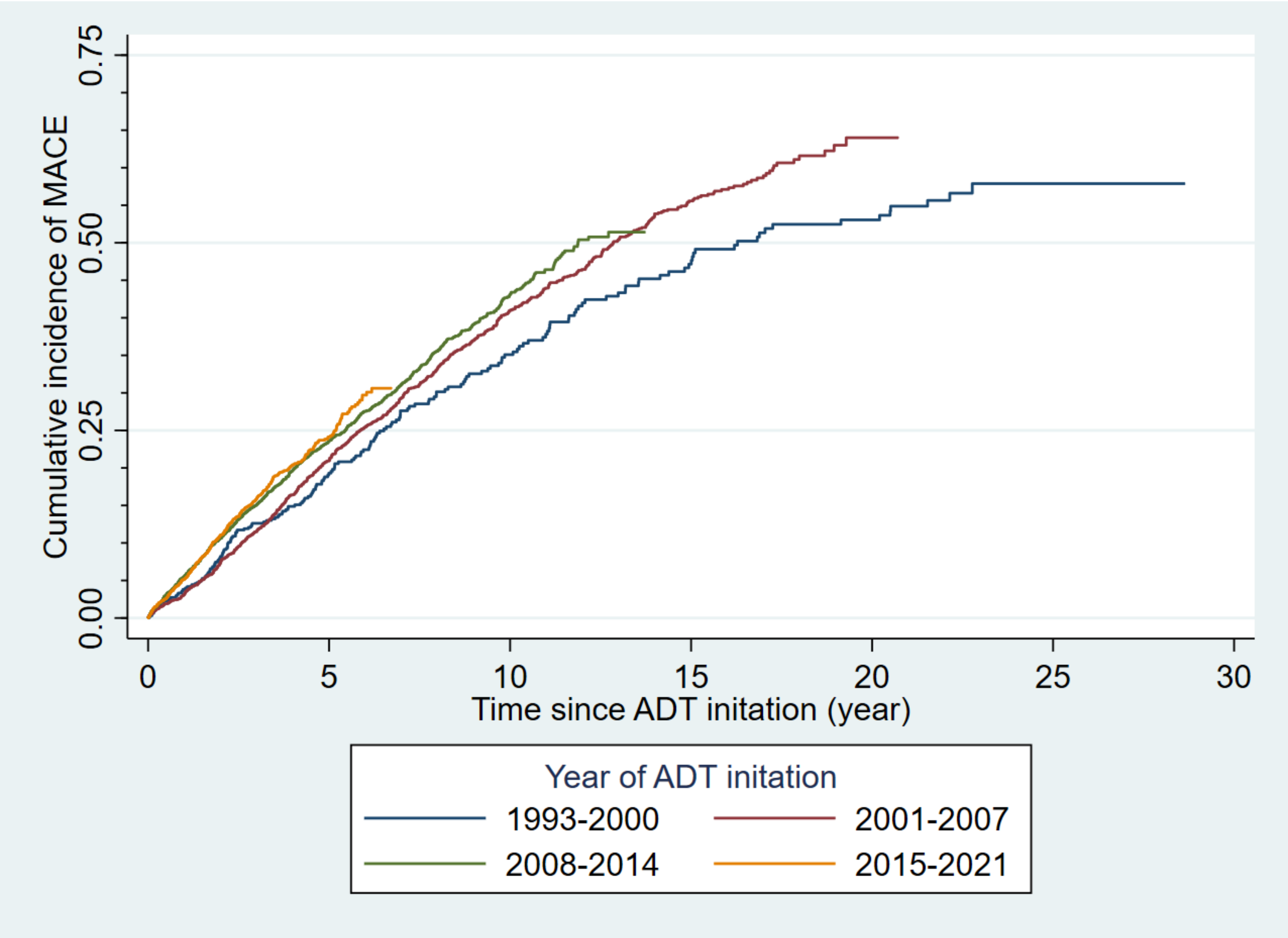

**Supplementary Figure 2.** Kaplan-Meier curve showing the cumulative incidence of all-cause mortality without any restriction on the follow-up duration. ADT, androgen deprivation therapy.

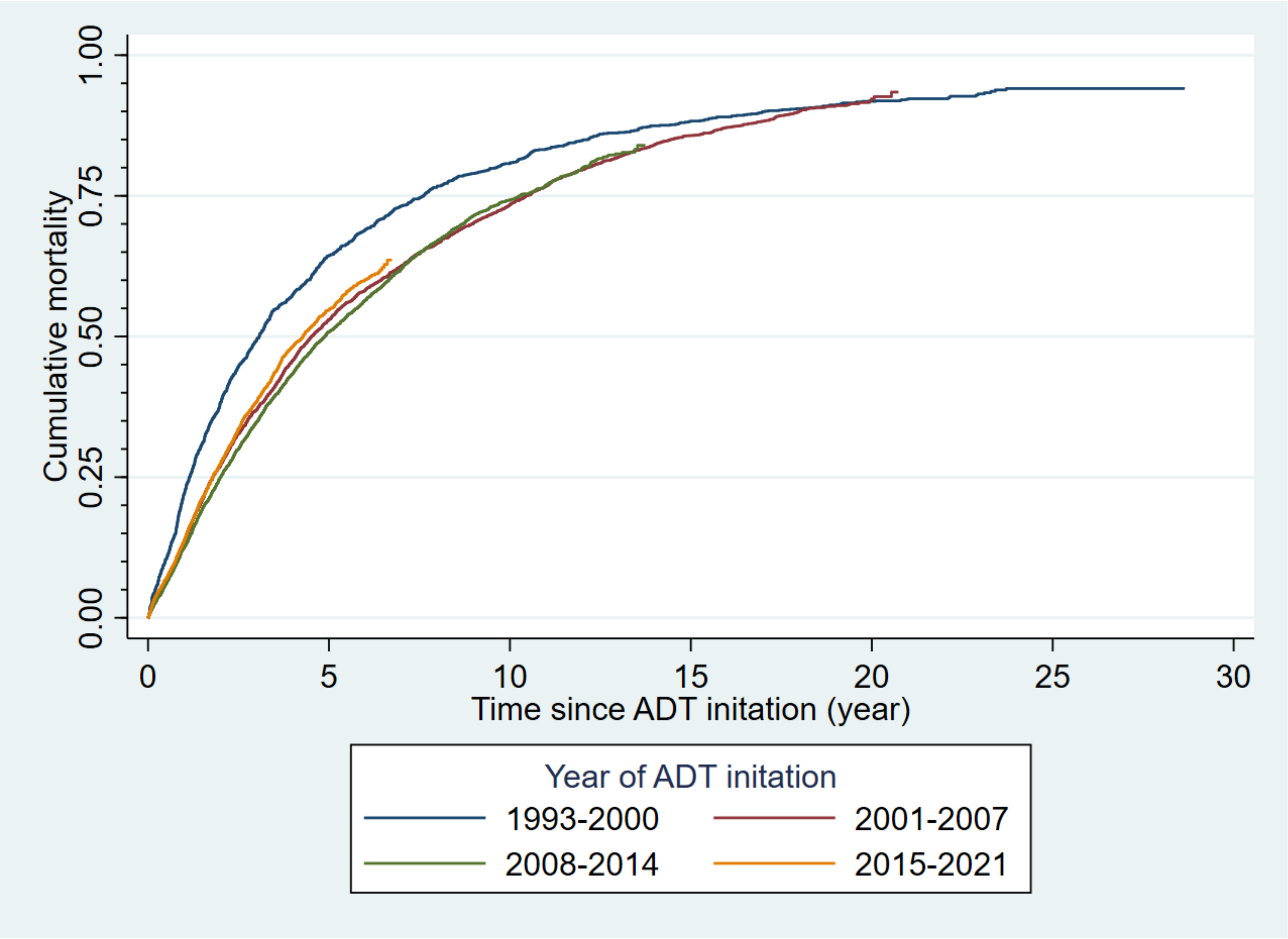

Supplement: Supplementary file 1 — Supplementary Material [file 41416_2023_2271_MOESM1_ESM.pdf]
